# Distinct Progression and Efficacy of First-Line Osimertinib Treatment According to Mutation Subtypes in Metastatic NSCLC Harboring EGFR Mutations

**DOI:** 10.1016/j.jtocrr.2024.100636

**Published:** 2024-01-18

**Authors:** Yuki Takeyasu, Tatsuya Yoshida, Ken Masuda, Yuji Matsumoto, Yuki Shinno, Yusuke Okuma, Yasushi Goto, Hidehito Horinouchi, Noboru Yamamoto, Yuichiro Ohe

**Affiliations:** a Department of Thoracic Oncology, National Cancer Center Hospital, Tokyo, Japan; b Department of Thoracic Oncology, Kansai Medical University Hospital, Osaka, Japan

**Keywords:** Non–small cell lung cancer, Epidermal growth factor receptor, EGFR TKI, Osimertinib, Central nervous system

## Abstract

**Introduction:**

Osimertinib (OSI), a third-generation EGFR tyrosine kinase inhibitor, is the standard treatment for patients with naive EGFR-mutant NSCLC. Nevertheless, information on how the mutation subtype affects disease progression after the failure of OSI treatment is scarce.

**Methods:**

We retrospectively reviewed patients with EGFR-mutant NSCLC who received OSI as a first-line treatment between April 2015 and December 2021.

**Results:**

This study included 229 patients. The objective response rate was 71%, with intracranial and extracranial response rates of 71% and 90%, respectively. The median progression-free survival was 23.3 mo (95% confidence interval [CI]: 19.6–26.7), and the median overall survival was 33.7 mo (95% CI: 31.3–58.6). Multivariate analysis revealed that the EGFR exon 21 L858R point mutation (L858R) (hazard ratio [HR] = 1.56, 95% CI: 1.04–2.34, *p* = 0.0328) and liver metastasis (HR = 2.63, 95% CI: 1.53–4.49, *p* = 0.0004) were significant predictors of progression-free survival in OSI treatment. The concomitant disease progression involving the central nervous system metastasis was significantly more common in patients with L858R (*p* = 0.048), whereas concomitant disease progression involving primary lesions was significantly more common in patients with exon 19 deletion mutation (*p* = 0.01). In addition, the probability of disease progression over time was higher for L858R compared with that for exon 19 deletion mutation, in patients with central nervous system metastasis (log-rank test, *p* = 0.027).

**Conclusions:**

The mutation subtype had an impact not only on the clinical outcome of the first-line OSI treatment but also on progression patterns after OSI treatment in patients with NSCLC harboring EGFR mutations.

## Introduction

EGFR mutations are the most common targetable mutations in patients with NSCLC.^1^ EGFR tyrosine kinase inhibitors (TKIs) have revolutionized the treatment of patients with NSCLC harboring EGFR mutations, especially sensitizing EGFR mutations such as exon 19 deletions (19del) and exon 21 L858R point mutations (L858R).^2^, ^3^, ^4^, ^5^, ^6^, ^7^ These common somatic mutations account for approximately 85% of EGFR gene alterations.^8^ Multiple EGFR TKIs have been established as the standard treatment for naive patients with NSCLC harboring EGFR-sensitizing mutations.

Osimertinib (OSI), a third-generation EGFR TKI, was developed for the treatment of patients with EGFR exon 20 T790M mutation-positive NSCLC, after the failure of the first- and second-generation EGFR TKIs.^9^^,^^10^ In the FLAURA trial, OSI was found to have a significant prolongation of progression-free survival (PFS) and overall survival (OS) and reduced the incidence of central nervous system (CNS) metastasis, compared with the first-generation EGFR TKIs erlotinib and gefitinib, as the initial therapy for treatment-naive patients with advanced NSCLC harboring EGFR mutations.^11^, ^12^, ^13^ Although OSI was found to have a marked therapeutic effect, almost all patients treated with OSI experience disease progression after approximately 15 to 20 months.

Several studies have suggested that the mutation subtype and metastatic site at the baseline are the predictive factors for the efficacy of EGFR TKIs.^14^^,^^15^ In contrast, there is little information on disease progression patterns after the failure of the OSI treatment, according to the mutation subtype. In this study, we evaluated the predictive factors of OSI response and the differences in progression patterns for OSI treatment in previously untreated NSCLC harboring EGFR mutations, on the basis of the type of mutation.

## Materials and Methods

### Study Design and Patients

Patients with EGFR-mutant NSCLC who received OSI (80 mg daily) as the initial therapy between April 2015 and December 2021 at a single-center, National Cancer Center hospital, in Japan, were included in this study. Patients who switched to other EGFR TKIs owing to adverse events were excluded. The cutoff date was March 30, 2022. Data including patient characteristics such as sex, age, cancer stage, histological type, smoking status, type of EGFR mutations, programmed cell death-ligand 1 (PD-L1) expression, metastatic pattern, Eastern Cooperative Oncology Group performance status (ECOG PS), and clinically measurable laboratory biomarkers were retrospectively evaluated from the medical records. EGFR mutations were assessed after NSCLC diagnosis using liquid or tissue biopsies, which were subjected to either next-generation sequencing (Oncomine Dx Target test, Thermo Fisher Scientific, Waltham, MA), Cobas EGFR mutation test (Roche Molecular Systems, Pleasanton, CA), *therascreen* EGFR-mutant test (Qiagen, Hilden, Germany), and AmoyDx Pan Lung Cancer PCR Panel (Amoy Diagnostics Co., Ltd., Xiamen, People’s Republic of China). Mutations other than 19del and L858R were defined as uncommon mutations. Tumor PD-L1 expression was analyzed through immunohistochemical analysis using the PD-L1 immunohistochemistry 22C3 pharmDx assay (Agilent, Santa Clara, CA). This study was approved by the Institutional Ethics Committee of the National Cancer Center Hospital (2015-355) and was performed in accordance with the Declaration of Helsinki.

### Statistical Analysis

The tumor response to OSI was determined on the basis of the Response Evaluation Criteria for Solid Tumors, version 1.1.^16^ Comparisons of categorical variables were performed using Fisher’s exact test or the chi-square test. PFS and OS were estimated using the Kaplan–Meier method, and comparisons were performed with the log-rank test. PFS was defined as the duration from the date of administration of the first dose of OSI to disease progression or death. OS was defined as the period between the administration of the initial dose of OSI and death. Both data were censored at the last follow-up visit. Cox proportional hazards models were used to analyze hazard ratios (HRs) and 95% confidence intervals (CIs) through univariate and multivariate analyses. On the basis of previous reports, age (≥70 y), sex, smoking status, ECOG PS (≥2), type of EGFR mutation, PD-L1 tumor proportion score (≥50%), and liver, bone, adrenal, and CNS metastases were selected as covariates.^14^^,^^17^, ^18^, ^19^, ^20^, ^21^ Competing risk methods were used to analyze the cumulative incidence of CNS metastasis. Each progression event was defined as either CNS metastasis progression, any other progression, or death, and the data were censored when the earliest event occurred. A *p* value less than 0.05 was considered statistically significant. The analyses were performed using JMP Pro version 13.1.0 (SAS Institute, Cary, NC).

## Results

### Patient Characteristics

Baseline patient characteristics are summarized in Table 1. A total of 229 patients with advanced NSCLC harboring EGFR mutations received OSI treatment; the median age was 67 years (range: 28–87 y) and 154 (68%) of the patients were female. More than half of the patients were nonsmokers (68%) and most of the patients had adenocarcinoma (97%). ECOG PS greater than or equal to 2 was observed in 27 patients (12%). The most common EGFR mutation was 19del (n = 125, 55%), followed by L858R (n = 88, 38%). Uncommon EGFR mutations included the following: L861Q (n = 8), S7681I (n = 3), G719X (n = 2), G719A (n = 1), G719C+E709A (n = 1), and G719X+S768I (n = 1). Patient characteristics for each type of EGFR mutation assessed in this study are presented in Supplementary Table 1 (which reveals baseline patient characteristics by type of EGFR mutation). At the initiation of OSI treatment, 73 patients (32%) had CNS metastasis. Of these patients, 26 had prior local treatments in the brain; 11 had received whole-brain radiation therapy whereas 12 had received stereotactic radiotherapy. At the data cutoff, 118 patients were still receiving the OSI treatment.

**Table 1:** Baseline Patient Characteristics

Patient Characteristics	All Patients (N = 229)
Age, median (range), y	67 (2–87)
Sex, n (%)	
Female	154 (68)
Smoking history, n (%)	
Never	156 (68)
Current or former	73 (32)
Histopathology, n (%)	
Adenocarcinoma	222 (97)
Others	7 (3)
Stage at diagnosis, n (%)	
III–IV	141 (62)
Recurrence	88 (38)
ECOG performance states, n (%)	
0	84 (37)
1	118 (52)
2	27 (11)
Mutation type, n (%)	
19del	125 (55)
L858R	88 (38)
Uncommon mutations	16 (7)
PD-L1 TPS, n (%)	
≥50%	24 (10)
1%–49%	61 (27)
<1%	108 (47)
Unknown	36 (16)
Site of metastasis, n (%)	
Pulmonary	77 (34)
Pleural dissemination	74 (32)
Liver metastasis	31 (14)
Bone metastasis	108 (47)
Adrenal metastasis	16 (7)
Brain metastasis	72 (32)
Prior SRS	12
Prior WBRT	11
Prior surgery	3

19del, exon 19 deletion; ECOG, Eastern Cooperative Oncology Group; L858R, L858R point mutation; PD-L1, programmed death-ligand 1; SRT, stereotactic radiosurgery; TPS, tumor proportion score; WBRT, whole-brain radiation therapy.

### Clinical Outcomes of OSI in Patients With Advanced NSCLC

The median follow-up duration was 19.7 mo (interquartile range: 13.8–27.5). In all patients who underwent OSI treatment, the objective response rate (ORR) was 71% (95% CI: 64.1–75.8), the disease control rate was 89% (95% CI: 84.4–92.5), and the intracranial (IC) ORR was 90% (95% CI: 80.2–95.4) (Supplementary Table 2, which reveals activity of OSI). The overall concordance between IC and extracranial (EC) response to OSI treatment was 89%. Among the patients with baseline CNS metastasis, 51 (84%) exhibited both IC and EC response whereas three (5%) exhibited neither. The patients with 19del had a significantly higher response than that in patients with L858R (79% versus 60%, *p* = 0.003). IC ORR was 97% with 19del versus 79% with L858R (*p* = 0.03). In the 19del, 42% of the patients exhibited an IC complete response, whereas only 25% of the patients in the L858R exhibited an IC complete response (Supplementary Table 3, which reveals activity of OSI by type of EGFR mutation).

**Figure 1:**
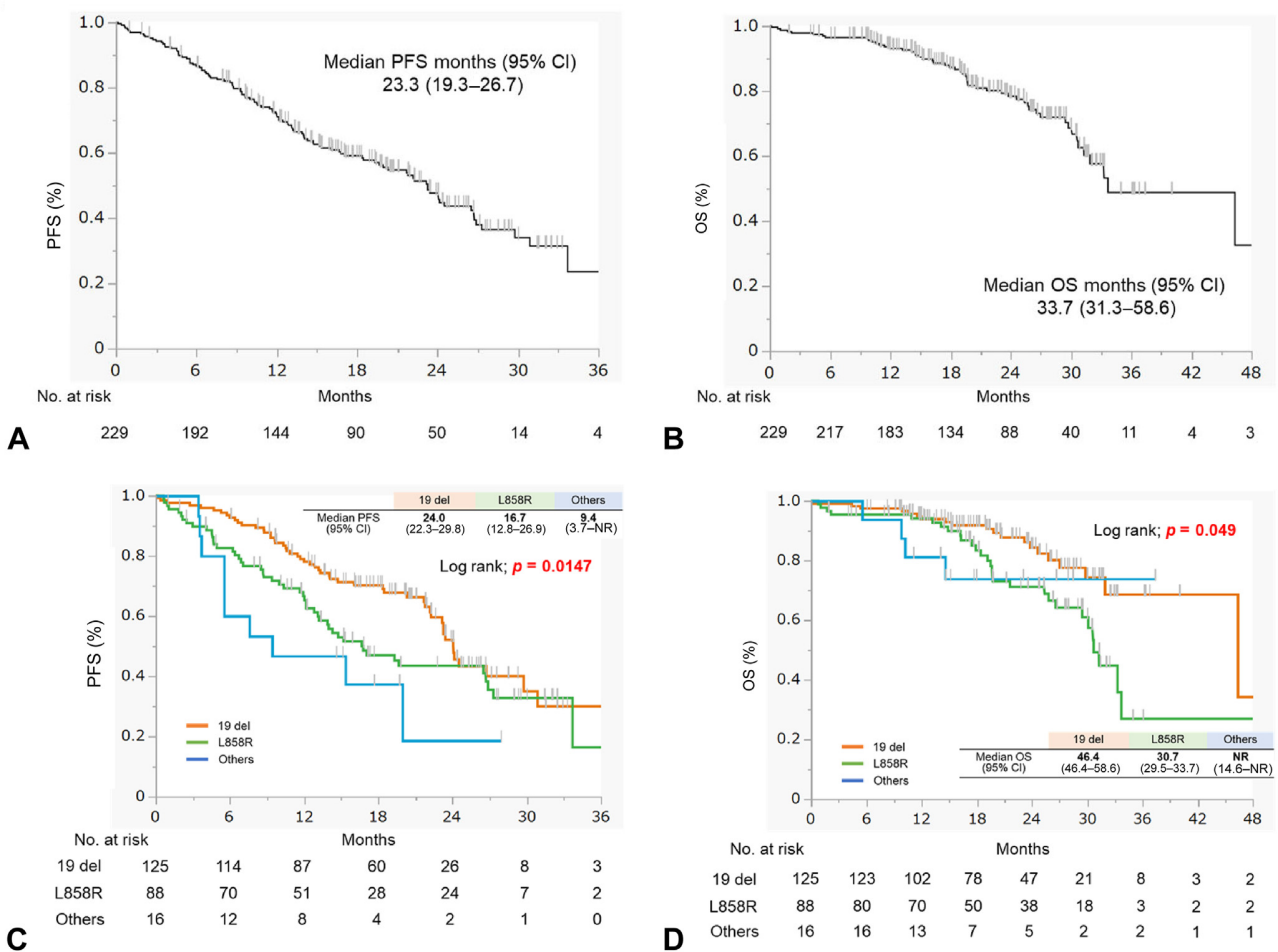
Efficacy of osimertinib treatment in patients with untreated NSCLC harboring EGFR mutations; (*A*) PFS for all patients and (*B*) OS for all patients. (*C*) PFS and (*D*) OS by type of EGFR mutation. 19del, exon 19 deletion; CI, confidence interval; L858R, L858R point mutation; OS, overall survival; PFS, progression-free survival.

The median PFS and OS were 23.3 mo (95% CI: 19.3–26.7) and 33.7 mo (95% CI: 31.3–58.6), respectively (Fig. 1*A* and *B*). The median PFS for 19del, L858R, and uncommon mutations was 24.0 mo (95% CI: 22.3–29.8), 16.7 mo (95% CI: 12.8–26.9), and 9.4 mo (95% CI: 3.7–not evaluated), respectively (Fig. 1*C*). The median OS for 19del, L858R, and uncommon mutations was 46.4 mo (95% CI: 46.4–58.6), 30.7 mo (95% CI: 29.5–33.7), and not reached (95% CI: 14.6–not reported), respectively (Fig. 1*D*).

### OSI Activity According to the Metastatic Sites and EGFR Mutation Subtype

**Figure 2:**
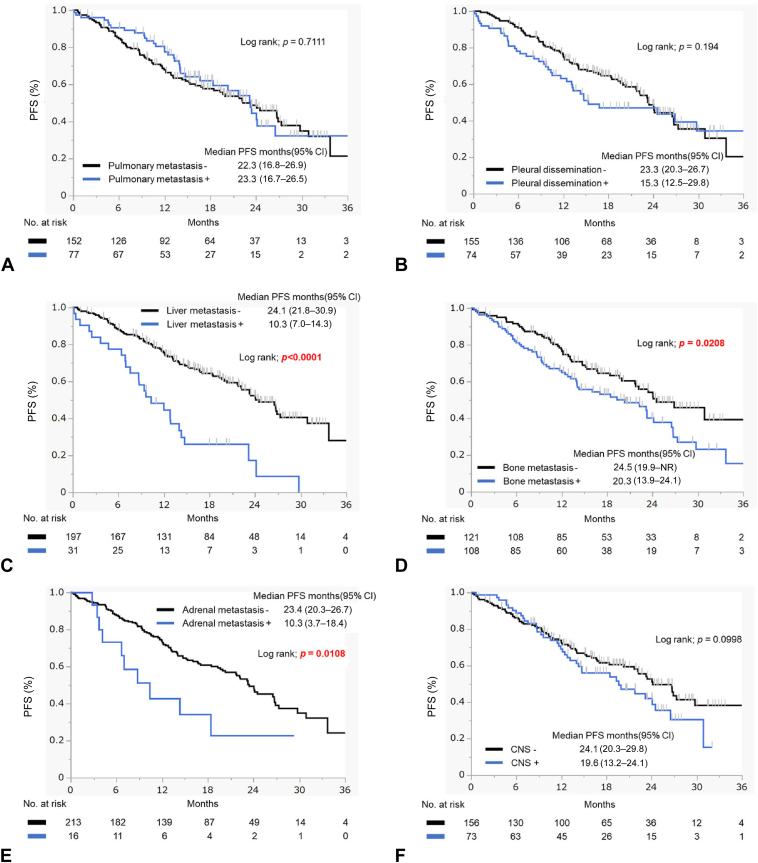
Efficacy of osimertinib treatment according to the metastatic sites. PFS for patients with and without (*A*) pulmonary metastasis, (*B*) pleural effusion, (*C*) liver metastasis, (*D*) bone metastasis, (*E*) adrenal metastasis, and (*F*) central nervous system metastasis. CI, confidence interval; NR, not reported; PFS, progression-free survival.

According to the metastatic sites, the median PFS in patients with liver, bone, or adrenal metastases was significantly shorter than in those without them, whereas there was no difference in the PFS between patients with and without brain metastasis (19.6 mo versus 24.1 mo; HR = 1.380; 95% CI: 0.94–2.04, *p* = 0.1015). Similar results were observed for pleural effusions (Fig. 2*A* to *F*). The multivariate analysis revealed that patients with L858R exhibited a significantly shorter PFS than those with 19del (HR = 1.56, 95% CI: 1.04–2.34, *p* = 0.0328) and patients with liver metastasis also exhibited a shorter PFS (HR = 2.63, 95% CI: 1.53–4.49, *p* = 0.0004) (Table 2).

**Table 2:** Univariate and Multivariate Analyses of Progression-Free Survival Using Cox Regression Models

Factor	Univariate Analysis	Multivariate Analysis		*p* Value
	Hazard Ratio	Hazard Ratio	95% CI	
Age (71≤/<70)	1.33	1.32	0.88–1.97	0.18
ECOG PS (2≤/0–1)	1.60	1.26	0.70–2.28	0.45
Sex, male/female	1.07	1.13	0.73–1.73	0.59
Smoking status (current or former/never)	1.20	1.17	0.75–1.82	0.48
EGFR mutation (L858R/19del)	1.44	1.56	1.04–2.34	0.03
PD-L1 TPS (50%≤/<50%^a^)	1.61	1.58	0.87–2.85	0.113^a^
Liver metastasis (±)	3.11	2.63	1.53–4.49	0.0004
Bone metastasis (±)	1.56	1.25	0.81–1.91	0.31
Adrenal metastasis (±)	1.99	1.80	0.88–3.68	0.10
CNS metastasis (±)	1.40	1.21	0.79–1.86	0.37

19del, exon 19 deletion; CI, confidence interval; CNS, central nervous system; ECOG, Eastern Cooperative Oncology Group; L858R, L858R point mutation; PD-L1, programmed death-ligand 1; PS, performance status; TPS, tumor proportion score.

a The statistical test was between PD-L1 TPS greater than or equal to 50% and other factors (including unknown).

In addition, we evaluated the impact of EGFR mutation subtypes (19del versus L858R) on PFS according to the metastatic spread at the baseline (Supplementary Fig 1*A* to *F*). Interestingly, in patients without brain metastasis at baseline, there was no difference in PFS between the patients with 19del and L858R (24.0 mo versus 26.7 mo; HR = 1.26, 95% CI: 0.77–2.07, *p* = 0.3479), whereas, in patients with brain metastasis, PFS was significantly shorter in patients with L858R compared with that in patients with 19del (12.8 mo versus 24.1 mo; HR = 2.13, 95% CI: 1.10–4.12, *p* = 0.0251).

### Distinct Progression Patterns During OSI According to EGFR Mutation Subtype

**Figure 3:**
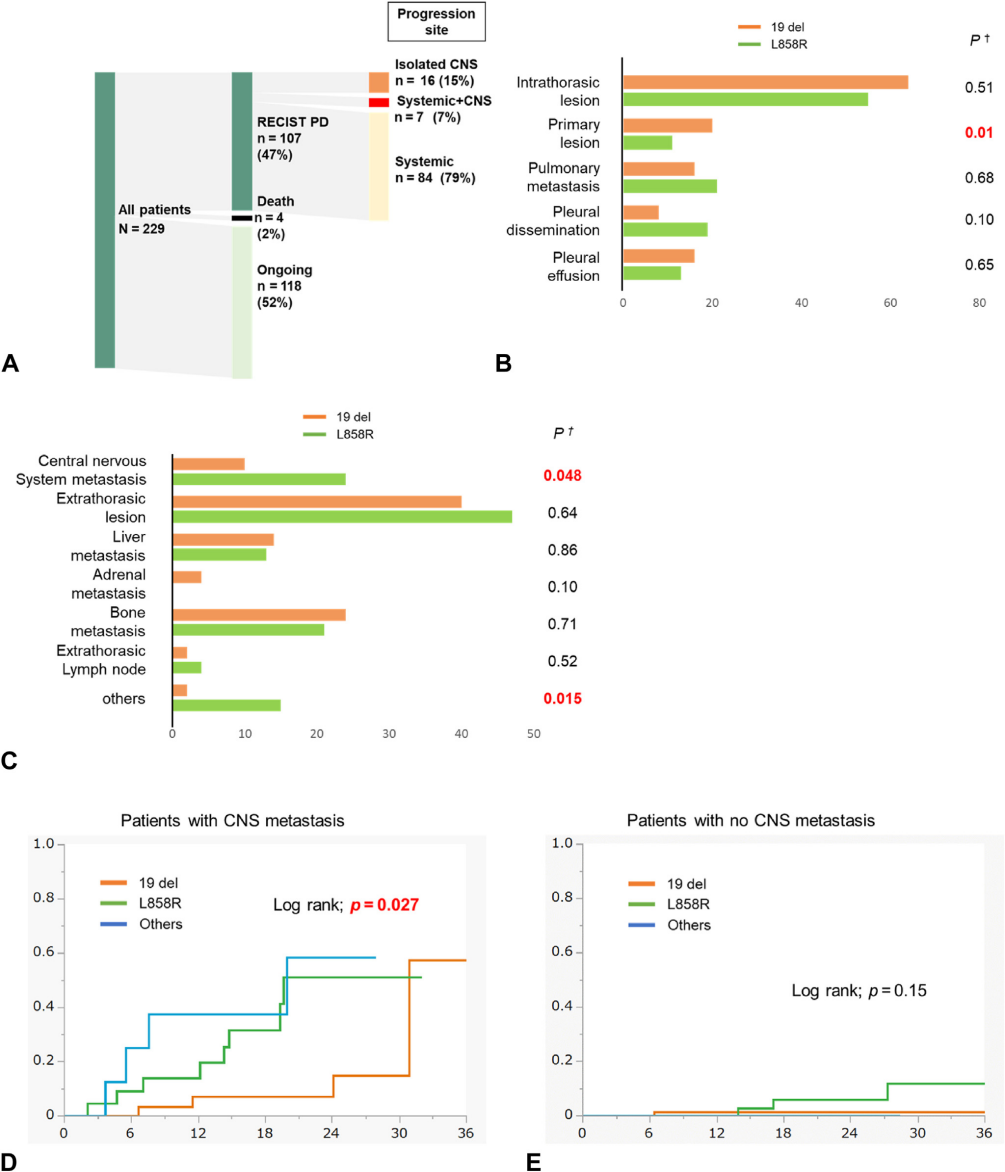
(*A*) Osimertinib administration status and disease progression pattern after osimertinib treatment failure. Frequency of progression pattern in (*B*) intrathoracic and (*C*) extrathoracic sites. Cumulative incidence of disease progression by type of EGFR mutation (*D*) in patients without CNS metastasis and (*E*) in patients with CNS metastasis. The statistical test was the probability of CNS metastasis progression between the mutation types exon 19 deletions and L858R point mutations. CNS, central nervous system.

Progression patterns are summarized in Figure 3*A* to *C*. During the course of the treatment, 107 patients exhibited progression of disease and 67 of them (63%) received subsequent treatment. The common sites of disease progression included intrathoracic lesions (60%), extrathoracic lesions (43%), and the CNS (21%). The concomitant disease progression involving CNS metastasis was significantly more common in patients with L858R (*p* = 0.048), whereas concomitant disease progression involving the primary lesion was significantly more common in patients with 19del (*p* = 0.01). Patients with CNS metastasis at the beginning of the treatment exhibited more frequent CNS metastasis progression than those without it (40% versus 6%, *p* < 0.0001). In patients with CNS metastasis (regardless of prior radiotherapy) at the baseline, the probability of CNS metastasis progression was higher over time for those with L858R compared with that in those with 19del (log-rank test; *p* = 0.027) (Fig. 3*D*). In contrast, in patients without CNS metastasis, there was no significant difference in the probability of CNS metastasis progression between those with 19del and L858R (log-rank test; *p* = 0.15) (Fig. 3*E*).

## Discussion

We evaluated the clinical outcomes of OSI treatment in previously untreated patients with NSCLC harboring EGFR mutations and identified the metastatic status and mutation subtypes that affected the clinical outcomes of OSI treatment. In addition, we observed that during OSI treatment, concomitant CNS metastasis progression was more common in patients with L858R, compared with that in patients with 19del. Our data suggest that the treatment strategy should be developed on the basis of the mutation subtype and metastatic spread.

Our data reveal that the median PFS and OS in patients treated with OSI as first-line therapy were 23.3 and 33.7 mo, respectively, and PFS in OSI-treated patients with L858R and uncommon mutations was significantly shorter compared with that for patients with 19del. These results are in accordance with those of the FLAURA study (19del: 21.4 mo; L858R: 14.4 mo)^12^^,^^13^ and other studies.^14^^,^^15^^,^^19^ In general, EGFR TKIs, including OSI, are more effective in patients with 19del than in those with L858R.^22^ One of the reasons for the difference in the efficacy of EGFR TKIs on 19del and L858R is the location of L858R at a distance from the ATP binding site^23^ and 19del being a structural change resulting from the deletion of an essential residue in the alpha-helix of the tyrosine kinase domain.^24^ Second, although ligand-less dimerization in the kinase domain is common owing to oncogenic activity, the activity of L858R is dimerization dependent, whereas that of 19del is dimerization independent.^25^ Third, the postdimerization autophosphorylation sites in 19del and L858R are different, as are the subsequent downstream signaling pathways.^26^

With respect to uncommon mutations, preclinical data revealed that OSI was active against most uncommon mutations, except for the exon 20 insertion mutation.^27^ Uncommon mutations have heterogeneous molecular alterations in exons from 18 to 21,^28^ and differences in the clinical efficacy of OSI depend on the type of uncommon mutations.^29^^,^^30^ A recent detailed study identified that a structure and function-based approach predicts patient clinical outcome and drug sensitivity in EGFR mutations, including uncommon mutations.^31^ Our results on uncommon mutations are consistent with those of two previous studies (median PFS: 8.2 mo and 9.5 mo).^29^^,^^30^

We also evaluated how the metastatic sites at the baseline and progression sites affect the clinical outcomes of first-line OSI treatment in patients with naive EGFR-mutant NSCLC. The presence of liver metastasis affected the clinical outcomes of OSI treatment the most, compared with other metastatic sites, which is in accordance with previous reports.^14^^,^^15^ A poor clinical outcome in patients with EGFR-mutant NSCLC with liver metastasis has been reported not only in patients treated with OSI but also in those treated with other first- and second-generation EGFR TKIs. Previous reports have revealed that the tumor microenvironment of liver metastasis has an increased expression of the vascular endothelial growth factor,^32^ and in EGFR-mutant NSCLC, activated EGFR signaling drives vascular endothelial growth factor expression in a hypoxia-independent manner.^33^ These contribute to resistance to EGFR TKIs in EGFR-mutant NSCLC with liver metastasis.

Progression patterns during EGFR TKI treatment of patients with advanced NSCLC harboring EGFR mutations are widely diverse. Importantly, approximately half of the patients with EGFR-mutant NSCLC develop brain metastasis,^34^ whereas up to 40% develop CNS metastasis over the course of the disease after the initial response to EGFR TKI therapy.^35^ Therefore, we evaluated the efficacy of OSI treatment against CNS metastasis and distinct progression patterns on the basis of the type of mutation. Interestingly, in patients with CNS metastasis, OSI treatment was less effective against L858R than 19del (12.8 mo versus 24.1 mo, *p* = 0.02). In contrast, in patients without CNS metastasis, there was no significant difference in the efficacy of OSI treatment between 19del and L858R (24.0 mo versus 26.7 mo, *p* = 0.35). In addition, regardless of the presence or absence of CNS metastasis at baseline, CNS metastasis was a significantly more common progression site in patients with L858R compared with that in patients with 19del. In patients without CNS metastasis at baseline, there was no significant difference in the probability of progression in those with 19del and L858R. These results may have been influenced by the shorter PFS and lower IC response of OSI treatment in patients with NSCLC with L858R, compared with that in patients with 19del. In this study, patients with 19del received more brain radiotherapy for brain metastases compared with those with L858R (46% versus 11%, *p* = 0.0023), suggesting a synergistic efficacy of brain radiotherapy and EGFR TKI as another reason for the distinct progression patterns. Patients with 19del have more brain lesions and smaller brain edema than those of EGFR wild type, which would have potentially led to radiotherapy.^36^ Radiotherapy enhances drug penetration of the blood–brain barrier and consequently increases the effective concentration of EGFR TKI.^37^ Indeed, previous reports revealed that combination therapy with radiotherapy and EGFR TKI was associated with better outcomes than those achieved with TKI alone in EGFR mutation-positive NSCLC with brain metastases.^38^^,^^39^ The biological rationale for differences in CNS metastasis development between 19del and L858R remains unclear owing to the limited insight into the genetic makeup of metastasis. The variation in resistance mutations and differences in co-occurring mutations may define the risk of CNS metastasis progression. Indeed, although the association between primary lesions and CNS metastasis with respect to EGFR mutations has been proven,^40^ the emergence of the EGFR exon 20 T790M resistance mutation in CNS metastasis is comparatively rare.^41^, ^42^, ^43^ Furthermore, the occurrence of EGFR exon 20 T790M-resistant mutations may be less common in tumors with concurrent TP53 mutations,^44^ and some of these concomitant mutations, including TP53 mutations, affect the inferior clinical outcome of EGFR TKI-treated patients.^45^ Our data provide a rationale for considering additional treatment strategies to prevent or delay the development of CNS metastasis, on the basis of the EGFR mutation subtype.

This study had several limitations. First, it was a retrospective, single-center study on the Japanese population. Second, the intervals for assessing radiological treatment responses depended on the treating physician, allowing for potential bias. Nevertheless, all patients underwent scheduled follow-up and radiological assessment. Third, resistance mutations and co-occurring mutations could not be evaluated because of the difficulty in collecting the tissue after OSI treatment failure. Finally, we were unable to assess the initial resistance mutations involved in the efficacy of OSI treatment, nor were we able to evaluate the biology of the cancer resistance.

In conclusion, our findings elucidate the metastatic patterns and type of EGFR mutations associated with the clinical outcomes of first-line OSI therapy and suggest that distinct progression patterns dependent on the type of EGFR mutation may be important in determining the treatment strategies in patients with EGFR-mutant NSCLC.

## CRediT Authorship Contribution Statement

**Yuki Takeyasu:** Study concepts, Study design, Data acquisition, Quality control of data and algorithms, Data analysis and interpretation, Statistical analysis, Manuscript preparation, Manuscript editing, Manuscript review.

**Tatsuya Yoshida:** Study concepts, Study design, Quality control of data and algorithms, Data analysis and interpretation, Statistical analysis, Manuscript preparation, Manuscript editing, Manuscript review.

**Ken Masuda:** Manuscript review.

**Yuji Matsumoto:** Manuscript review.

**Yuki Shinno:** Manuscript review

**Yusuke Okuma:** Manuscript review.

**Yasushi Goto:** Manuscript review.

**Hidehito Horinouchi:** Manuscript review.

**Noboru Yamamoto:** Manuscript review.

**Yuichiro Ohe:** Manuscript review.

## Disclosure

Dr. Yoshida reports receiving grants and personal fees from AstraZeneca and Bristol-Myers Squibb and grants from 10.13039/100008373Takeda. Dr. Matsumoto reports receiving grants from the National Cancer Center Research and Development Fund, Grant-in-Aid for Scientific Research on Innovative Areas, Hitachi, Ltd., and Hitachi High-Technologies, and personal fees from Olympus, AstraZeneca, and Novartis. Dr. Shinno reports receiving grants and personal fees from Ono Pharmaceutical; grants from 10.13039/100020707Janssen Pharmaceutical, Japan Clinical Research Operations, 10.13039/100009954Taiho Pharmaceutical, and 10.13039/100004326Bayer; and personal fees from AstraZeneca, Bristol-Myers Squibb, Chugai Pharmaceutical, and Eli Lilly Japan. Dr. Okuma reports receiving grants and personal fees from AstraZeneca; personal fees from Boehringer Ingelheim, Chugai Pharmaceutical Co. Ltd., Daiichi Sankyo, Eisai, Eli Lilly, Merck Sharp & Dohme, Ono Pharmaceutical Co. Ltd., Taiho Pharmaceutical Co. Ltd., and Takeda Pharmaceutical Co. Ltd. Dr. Goto reports receiving grants and personal fees from Pfizer, Eli Lilly, Ono, Bristol-Myers Squibb, and Novartis; grants from AZK, 10.13039/100006483AbbVie, 10.13039/100004336Novartis, 10.13039/100019271Kyorin, 10.13039/501100022274Daiichi Sankyo, and Preferred Network; and personal fees from Chugai, Taiho, Boehringer Ingelheim, Merck Sharp & Dohme, Merck, and Thermo Fisher. Dr. Horinouchi reports receiving grants and personal fees from Bristol-Myers Squibb, Merck Sharp & Dohme, Chugai, Taiho, AstraZeneca, Eli Lilly, and Ono, and grants from 10.13039/501100004948Astellas, Merck Serono, and 10.13039/100008067Genomic Health. Dr. Yamamoto reports receiving grants, consulting fees, and personal fees from Eisai and Chugai; grants and personal fees from Ono and Daiichi Sankyo; grants and consulting fees from Takeda, Boehringer Ingelheim, Cmic, and Merck; and grants from Astellas, Taiho, 10.13039/100002491Bristol-Myers Squibb, 10.13039/100004319Pfizer, 10.13039/100004336Novartis, Eli Lilly, AbbVie, 10.13039/100004326Bayer, 10.13039/501100004095Kyowa Kirin, Janssen Pharma, 10.13039/100009947Merck Sharp & Dohme, 10.13039/100004330GlaxoSmithKline, Sumitomo Pharma, Chiome Bioscience, 10.13039/100019120Otsuka, Carna Biosciences, Genmab, 10.13039/501100005612Shionogi, 10.13039/100019943TORAY, 10.13039/501100001691KAKEN, 10.13039/100004325AstraZeneca, InventisBio, and 10.13039/100020413Rakuten Medical. Dr. Ohe reports receiving grants and personal fees from AstraZeneca, Chugai, Eli Lilly, Ono, Bristol-Myers Squibb, Pfizer, and Taiho; grants from 10.13039/100019271Kyorin, Dainippon-Sumitomo, Novartis, Takeda, 10.13039/100016288Kissei, Daiichi Sankyo, Janssen, and LOXO; and personal fees from Boehringer Ingelheim, Bayer, Merck Sharp & Dohme, Nippon Kayaku, and Kyowa Hakko Kirin. Drs. Takeyasu and Masuda declare no conflict of interest.
