# Supplementary material for: Distinct Progression and Efficacy of First-Line Osimertinib Treatment According to Mutation Subtypes in Metastatic NSCLC Harboring EGFR Mutations
Source: JTO Clin Res Rep. 2024 Jan 18;5(2):100636. doi: 10.1016/j.jtocrr.2024.100636 (PMC10867446; doi:10.1016/j.jtocrr.2024.100636)
Supplement: Supplementary Tables [file mmc1.docx]

Supplemental Table 1. Baseline patient characteristics by type of EGFR mutation.

| **Patient characteristics** | **ex19 del**  ***n*=125** | **L858R**  ***n*=88** | **Others**  ***n*=16** | ***P*-value*** |
| --- | --- | --- | --- | --- |
| **Age, median (range), years**  **Sex, *n* (%)**  Female  **Smoking history, *n* (%)**  Never  **Histopathology, *n* (%)**  Adenocarcinoma  **Stage at diagnosis, *n* (%)**  Recurrence  **ECOG performance states, *n* (%)**  0  1  2-  **PD-L1 TPS**  ≥50%  1–49%  <1%  Unknown  **Site of metastasis, *n* (%)**  Pulmonary  Pleural dissemination  Liver metastasis  Bone metastasis  Adrenal metastasis  Brain metastasis  Prior radiotherapy  SRS  WBRT  Prior surgery | 65 (32–87)  80 (65)  81 (65)  123 (98)  48 (38)  44 (35)  64 (51)  17 (14)  12 (10)  28 (22)  63 (50)  22 (18)  46 (37)  40 (32)  18 (15)  60 (48)  9 (7)  37 (30)  17  6  11  2 | 69 (28–87)  63 (72)  63 (72)  84 (95)  32 (36)  34 (39)  45 (51)  9 (10)  8 (9)  31 (35)  38 (43)  11 (13)  29 (33)  30 (34)  11 (13)  38 (43)  5 (6)  28 (32)  3  3  0  1 | 69 (33–78)  11 (69)  12 (75)  15 (94)  8 (50)  6 (38)  9 (56)  1 (6)  4 (25)  2 (13)  7 (44)  3 (19)  2 (13)  4 (25)  2 (13)  10 (63)  2 (13)  8 (50)  4  4  0  0 | 0.22  0.30  0.20  0.76  0.46**  0.90***  0.56  0.75  0.67  0.49  0.66  0.73  0.0023 |

ECOG: Eastern Cooperative Oncology Group; PD-L1: Programmed death-ligand 1; TPS: Tumor proportion score; SRT: Stereotactic radiosurgery; WBRT: Whole-brain radiation therapy.

*The statistical test was between the mutation types 19 deletions and L858R point mutations. ** The statistical test was between performance status (PS) 0–1 and PS ≥2. ***The statistical test was between PD-L1 TPS ≥50% and others (including unknown).

Supplemental Table 2. Activity of osimertinib.

|  | **All patients** (***N*=229)** |
| --- | --- |
| **Objective response rate (ORR). n (%)** | **162 (71)** |
| **Extracranial (EC). *n* (%)** | ***n*=229** |
| **Best overall response. *n* (%)**  CR  PR  Disease stability  **Clinical benefit**  EC-ORR  95% CI | 3 (1)  158 (69)  43 (19)  **70.7**  64.1–75.8 |
| **Measurable Intracranial (IC). *n* (%)** | ***n*=61** |
| **Best overall response. *n* (%)**  CR  Improvement  Disease stability  **Clinical benefit**  IC-ORR  95% CI | 20 (33)  35 (57)  6 (10)  **90.2**  80.2–95.4 |

CR: Complete response; PR: Partial response; CI: Confidence interval.

Supplemental Table 3. Activity of osimertinib by type of EGFR mutation.

|  | **exon 19 del**  ***n*=125** | **L858R**  ***n*=88** | **Others**  ***n*=16** | ***p*-value*** |
| --- | --- | --- | --- | --- |
| **Objective response rate (ORR). *n* (%)** | **97 (78)** | **53 (60)** | **9 (60)** | **0.003** |
| **Extracranial (EC). *n* (%)** | ***n*=125** | ***n*=88** | ***n*=16** |  |
| **Best overall response. *n* (%)**  CR  PR  Disease stability  **Clinical benefit**  EC-ORR  95% CI | 2 (2)  97 (78)  16 (13)  **99 (79)**  71.3–85.4 | 1 (1)  52 (59)  21 (24)  **53 (60)**  49.8–69.8 | 0  9 (56)  5 (38)  **9 (56)**  33.2–76.9 | **0.003** |
| **Measurable Intracranial (IC). *n* (%)** | ***n*=33** | ***n*=24** | ***n*=4** |  |
| **Best overall response. *n* (%)**  CR  Improvement  Disease stability  **Clinical benefit**  IC-ORR  95% CI | 14 (42)  18 (55)  1 (3)  **32 (97)**  84.7-99.5 | 6 (25)  13 (54)  5 (21)  **19 (79)**  59.5-90.8 | 0  4 (100)  0  **4 (100)** | **0.03** |

CR: Complete response; PR: Partial response; CI: Confidence interval. *The statistical test was between the mutation types 19 deletions and L858R point mutations.
