# Supplementary figures and images for: Distinct Progression and Efficacy of First-Line Osimertinib Treatment According to Mutation Subtypes in Metastatic NSCLC Harboring EGFR Mutations
Source: JTO Clin Res Rep. 2024 Jan 18;5(2):100636. doi: 10.1016/j.jtocrr.2024.100636 (PMC10867446; doi:10.1016/j.jtocrr.2024.100636)

## Slide 1
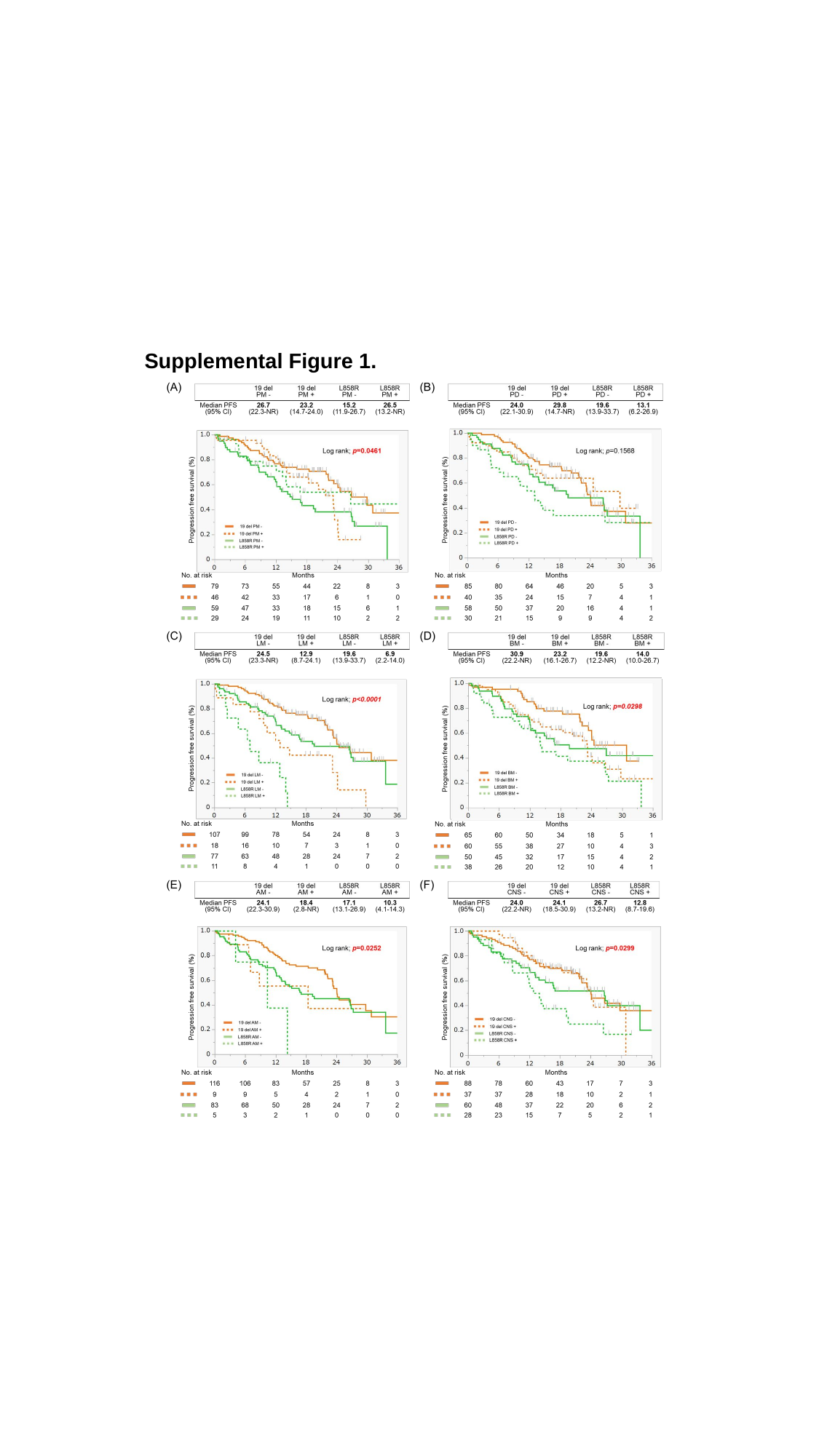

Supplemental Figure 1.

Supplement: Supplementary Figure [file mmc2.pptx]
